# Neural bases of Theory of Mind in children with autism spectrum disorders and children with conduct problems and callous-unemotional traits

**DOI:** 10.1111/desc.12167

**Published:** 2014-03-17

**Authors:** Elizabeth O'Nions, Catherine L Sebastian, Eamon McCrory, Kaylita Chantiluke, Francesca Happé, Essi Viding

**Affiliations:** 1 Developmental Risk & Resilience Unit, Clinical, Educational, and Health Psychology Research Department, University College LondonUK; 2 Department of Psychology, Royal Holloway, University of LondonUK; 3 Developmental Risk &Resilience Unit, Clinical, Educational, and Health Psychology Research Department, University College London,UK

## Abstract

Individuals with autism spectrum disorders (ASD) have difficulty understanding other minds (Theory of Mind; ToM), with atypical processing evident at both behavioural and neural levels. Individuals with conduct problems and high levels of callous-unemotional (CU) traits (CP/HCU) exhibit reduced responsiveness to others' emotions and difficulties interacting with others, but nonetheless perform normally in experimental tests of ToM. The present study aimed to examine the neural underpinnings of ToM in children (aged 10–16) with ASD (*N *=* *16), CP/HCU (*N *=* *16) and typically developing (TD) controls (*N *=* *16) using a non-verbal cartoon vignette task. Whilst individuals with ASD were predicted to show reduced fMRI responses across regions involved in ToM processing, CP/HCU individuals were predicted to show no differences compared with TD controls. The analyses indicated that neural responses did not differ between TD and CP/HCU groups during ToM. TD and CP/HCU children exhibited significantly greater medial prefrontal cortex responses during ToM than did the ASD group. Within the ASD group, responses in medial prefrontal cortex and right temporoparietal junction (TPJ) correlated with symptom severity as measured by the Autism Diagnostic Observation Schedule (ADOS). Findings suggest that although both ASD and CP/HCU are characterized by social difficulties, only children with ASD display atypical neural processing associated with ToM.

## Introduction

Theory of Mind (ToM) describes the ability to attribute mental states in order to explain or predict behaviour (Premack & Woodruff, 1978). Research indicates that individuals with autism spectrum disorders (ASD) have impairments in ToM (Baron-Cohen, Leslie & Frith, 1985; Senju, Southgate, White & Frith, 2009). For example, children with autism have difficulties attributing mental states such as beliefs or intentions to explain characters' actions or communication in simple stories (Baron-Cohen, O'Riordan, Jones, Stone & Plaisted, 1999; Happé, 1994; White, Hill, Happé & Frith, 2009). Social difficulties are mirrored by atypical neural processing, with most fMRI studies to date reporting reduced neural responses in adults and children with ASD relative to controls across a network of regions implicated in ToM (posterior superior temporal sulcus (pSTS)/temporoparietal junction (TPJ), medial prefrontal cortex (mPFC) and temporal poles) (e.g. Castelli, Frith, Happé & Frith, 2002; Lombardo, Chakrabarti, Bullmore & Baron-Cohen, 2011; Mason, Williams, Kana, Minshew & Just, 2008; Wang, Lee, Sigman & Dapretto, 2006).

In contrast, individuals with conduct problems and high levels of callous-unemotional (CU) traits (CP/HCU) appear to show intact ToM but reduced affective reactivity to others' emotions (Blair, 2005; Jones, Happé, Gilbert, Burnett & Viding, 2011; Schwenck, Mergenthaler, Keller, Zech, Salehi, Taurines, Romanos, Schecklmann, Schneider, Warnke & Freitag, 2011). Several studies have reported reduced psychophysiological reactivity to distress cues in children with CP/HCU (e.g. de Wied, van Boxtel, Matthys & Meeus, 2012; Kimonis, Frick, Munoz & Aucoin, 2007). Neuroimaging studies have found reduced amygdala response to fearful faces in children with CP/HCU (Jones, Laurens, Herba, Barker & Viding, 2009; Marsh, Finger, Fowler, Adalio, Jurkowitz, Schechter, Pine, Decety & Blair, 2013; Marsh, Finger, Mitchell, Reid, Sims, Kosson, Towbin, Leibenluft, Pine & Blair, 2008; Viding, Sebastian, Dadds, Lockwood, Cecil, De Brito & McCrory, 2012) and reduced responses to others' pain or distress across amygdala, anterior insula and dorsal/rostral anterior cingulate cortex (Lockwood, Sebastian, McCrory, Hyde, Gu, De Brito & Viding, 2013; Marsh *et al*., 2013; Sebastian, McCrory, Cecil, Lockwood, De Brito, Fontaine & Viding, 2012a). However, cognitive-experimental studies in adults with psychopathy and children with CP/HCU indicate intact ToM across a range of measures (Dolan & Fullam, 2004; Richell, Mitchell, Newman, Leonard, Baron-Cohen & Blair, 2003; Jones *et al*., 2011), although this has not yet been explored with fMRI.

Whilst behavioural evidence suggests that ToM is impaired in ASD but intact in CP/HCU, no study has directly compared the neural basis of ToM across these groups. Indeed, only one imaging study has directly compared ASD and anti-social traits, determining differential contributions to structural brain development in children, with only ASD traits associated with cortical thinning in superior temporal regions recruited during performance (Wallace, Shaw, Lee, Clasen, Raznahan, Lenroot, Martin & Giedd, 2012). Imaging methods can pick up subtle differences not always detectable at the cognitive or behavioural levels (e.g. Carter, Williams, Minshew & Lehman, 2012; Kana, Keller, Cherkassky, Minshew & Just, 2009), and as such provide a clearer picture of whether atypical processing associated with ToM is limited to ASD.

Here we used a cartoon-based vignette task (Sebastian, Fontaine, Bird, Blakemore, De Brito, McCrory & Viding, 2012b; Sebastian *et al*., 2012a) to explore the neural bases of ToM in ASD, CP/HCU and TD children. We compared responses during cartoons requiring ToM (understanding intentions) versus physical causality (PC) (understanding cause and effect without ToM demands). The CP/HCU and TD groups reported here contributed data to a previous paper (Sebastian *et al*., 2012a), which focused on a third, affective condition, tapping understanding of emotions within an intentional, narrative context. In the previous study, the focus was on the CP/HCU group and contrasting affective versus ToM conditions to examine affective processing in children with conduct problems. The current study focuses on the comparison of ASD and CP/HCU groups in ToM processing relative to the PC control condition. Since affective processing in an intentional context involves the integration of ToM and empathy-related processes (Shamay-Tsoory, 2011), and hence requires intact ToM, the affective condition was not analysed in the current study, since interpretation of any observed differences would be problematic given likely ToM deficits in the ASD group. Previous use of the present vignette task (Sebastian *et al*., 2012a, 2012b), comparing ToM versus PC conditions in typically developing populations has yielded reliable and replicable responses in the ‘mentalizing network’ comprising the pSTS extending to the TPJ, precuneus, temporal poles, and mPFC, in line with other studies using non-verbal cartoon stimuli (Brunet, Sarfati, Hardy-Bayle & Decety, 2000; Carter *et al*., 2012; Gallagher, Happé, Brunswick, Fletcher, Frith & Frith, 2000; Kana, Libero, Hu, Deshpande & Colburn, 2014; Völlm, Taylor, Richardson, Corcoran, Stirling, McKie, Deakin & Elliott, 2006).

Studies of ToM in adult ASD samples have reported reductions in mPFC response (Castelli *et al*., 2002; Happé, Ehlers, Fletcher, Frith, Johansson, Gillberg, Dolan, Frackowiak & Frith, 1996; Kana *et al*., 2009; Kennedy & Courchesne, 2008; Mason *et al*., 2008; Murdaugh, Shinkareva, Deshpande, Wang, Pennick & Kana, 2012; Watanabe, Yahata, Abe, Kuwabara, Inoue, Takano *et al*., 2012) and reductions or reduced selectivity of pSTS/TPJ (Castelli *et al*., 2002; Lombardo *et al*., 2011; Mason *et al*., 2008), pSTS (Pelphrey, Morris & McCarthy, 2005) and temporal poles (Castelli *et al*., 2002) relative to control participants. Several studies have reported negative correlations between autistic symptoms or ToM impairment and functional responses in pSTS (Kana *et al*., 2009; Pelphrey *et al*., 2005), mPFC (Kennedy & Courchesne, 2008) and right TPJ (Lombardo *et al*., 2011).

Three fMRI studies examining ToM in ASD have been conducted with child samples. One study, using static cartoon stimuli in children aged 7 to 16 with and without ASD, reported significantly reduced activation in mPFC, STS, left temporal pole, and precuneus during ToM (Carter *et al*., 2012). The two other studies have reported similar results, including negative correlations between autistic social symptoms and superior temporal responses (Wang *et al*., 2006), and between social responsiveness and medial PFC activation (Wang, Lee, Sigman & Dapretto, 2007).

This study aimed to test whether neural processing associated with ToM is abnormal in ASD in contrast to CP/HCU. Despite social deficits in both groups, they are rarely directly compared in the existing literature, and have never been compared with fMRI. To our knowledge, ours is also the first fMRI study to examine the neural correlates of ToM in children with CP/HCU, and the first to extend use of a non-verbal cartoon vignette task to a sample of children with ASD. Use of non-verbal vignettes to assay ToM is an advantage because individuals with ASD are reported to show impairments in deriving meaning from verbal stimuli (e.g. Randi, Newman & Grigorenko, 2010). In line with previous studies, we predicted reduced activation across the ‘mentalizing network’ in individuals with ASD compared to both CP/HCU and TD control groups. In particular, atypical responses were predicted in mPFC, pSTS/TPJ, and temporal poles in ASD. By contrast, based on cognitive-experimental evidence, we predicted intact responses in the CP/HCU group.

## Method

### Participants

Participants were a community sample of adolescent males (aged 10–16) who were typically developing (TD, *N *=* *16), had conduct problems plus callous unemotional traits (CP/HCU, *N *=* *16), or an autism spectrum disorder (ASD, *N *=* *16). Details of TD and CP/HCU participant recruitment are reported elsewhere (Sebastian *et al*., 2012a).

The ASD group were identified through their previous participation in research studies at King's College London or UCL. They were originally recruited from the community via schools and parent groups, and had a clinical diagnosis of autism or Asperger syndrome. They were assessed using the Autism Diagnostic Observational Schedule (Lord, Risi, Lambrecht, Cook, Leventhal, DiLavore, Pickles & Rutter, 2000) and, for nine participants, a full developmental interview (either the Autism Diagnostic Interview - Revised (ADI-R) or the 3di; Table S1) (Lord, Rutter & Couteur, 1994; Skuse, Warrington, Bishop, Chowdhury, Lau, Mandy & Place, 2004). For the remainder, developmental history was assessed using the Social Communication Questionnaire (based on the ADI; Eaves, Wingert, Ho & Mickelson, 2006). No cut-off for conduct problems or CU traits was imposed in the ASD group, but all ASD participants scored below the median for CU traits reported by Sebastian *et al*. (2012a), and ASD and TD groups did not differ on CP symptoms (from the Child and Adolescent Symptom Inventory-4R – Conduct Disorder subscale, CASI-CD; Gadow & Sprafkin, 2009). In line with previous studies (e.g. Bird, Silani, Brindley, White, Frith & Singer, 2010), participants who did not meet full criteria on all ASD assessment measures but had a clinical diagnosis were retained in the analyses. This allowed us to recruit sufficient numbers of high-functioning participants who could tolerate scanning. No participants were currently taking psychoactive medication, except for occasional melatonin (*N *=* *1), which was not used for 24 hours prior to scanning.

Participants with CP/HCU scored in the clinically relevant range on the CASI-CD subscale (> 2 at 10–14 years; > 5 at 15–16 years), and comprised the top 50% of the sample from Sebastian *et al*. (2012a) in terms of scores on the Inventory of Callous-Unemotional traits (ICU; Essau, Sasagawa & Frick, 2006). TD controls all scored below the clinical threshold for conduct problems and below 45 on the ICU. Parent/guardian data screening for psychiatric and neurological conditions, general psychopathology (including conduct problems and CU traits), and demographic data (including parent-defined ethnicity, handedness and socioeconomic status, based on National Statistics Socio-economic Classification NS-SEC coding; http://www.ons.gov.uk) was available for all participants and is displayed in Table1. To ensure that case groups were representative of ASD and CP/HCU, co-occurring symptoms of generalized anxiety disorder, major depression, substance abuse, or ADHD did not result in exclusion, but were measured using the CASI and their effects explored in the analyses. All participants completed the Wechsler Abbreviated Scale of Intelligence (2-subtest version;Wechsler, 1999), the Alcohol Use Disorders Identification Test (AUDIT; Saunders, Aasland, Babor, de la Fuente & Grant, 1993), and the Drug Use Disorders Identification Test (DUDIT; Berman, Bergman, Palmstierna & Schlyter, 2005).

**Table 1:** Demographic information

Demographic variables	Typically developing (*N* = 16)	CP/HCU (*N* = 16)	ASD (*N* = 16)	*p*-value	Difference
Age (years)	13.51 (1.65)	14.15 (1.88)	14.18 (1.63)	.47	
Socioeconomic status	2.70 (0.85)	3.19 (1.07)	2.69 (0.95)	.26	
Full-scale IQ	106.69 (12.67)	98.13 (11.98)	107.31 (13.23)	.08	
Verbal T score	56.94 (10.52)	49.13 (8.74)	55.25 (8.70)	.06	
Matrix reasoning T score	50.13 (8.61)	48.38 (9.27)	52.88 (9.94)	.39	
*Race/ ethnicity (N)*					
White	14	13	14		
Black	1	1	1		
Mixed race	1	2	1	.83	
*Handedness (N)*					
Right	11	14	15		
Left	4	2	1		
Ambidextrous	1	0	0	.14	
Inventory of Callous Unemotional traits (parent rated)	16.87 (5.72)	46.46 (7.02)	27.24 (8.99)	<.001	TD<ASD<CP/HCU
*Child and Adolescent Symptom Inventory (parent rated)*					
Conduct disorder symptoms	0.61 (0.85)	10.29 (5.45)	1.25 (1.39)	<.001	TD=ASD<CP/HCU
ADHD symptoms	9.88 (6.20	31.25 (9.09)	23.50 (9.91)	<.001	TD<ASD<CP/HCU
Generalised anxiety symptoms	3.75 (3.19)	8.13 (5.17)	9.38 (3.72)	.001	TD<ASD=CP/HCU
Major depressive symptoms	2.75 (1.98)	5.47 (3.34)	6.75 (4.48)	.006	TD<ASD=CP/HCU
Autism spectrum symptoms	1.40 (2.35)	4.27 (3.99)	13.88 (7.07)	<.001	TD<CP/HCU<ASD
Alcohol use and disorders	1.19 (1.76)	4.75 (7.26)	0.33 (0.62)	.02	ASD<TD=CP/HCU
Drug use and disorders	0.00 (0.00)	1.06 (2.62)	0.13 (0.50)	.11	

Nineteen children with ASD were scanned. Data from three ASD participants were excluded due to excessive motion, leaving a final ASD sample of 16 to be compared with the 16 TD and 16 CP/HCU participants described above. Written informed parental consent and written assent from participants was obtained. Groups were matched on age, IQ, SES, gender and handedness (see Table1).

### Experimental task

The task involved 30 cartoons, 10 each for ToM, physical causality (PC) and Affective ToM conditions. As noted in the introduction, the present study focuses on ToM and PC conditions only. Each cartoon was silent and static, and depicted two people in everyday scenarios (e.g. pouring a drink; going for a walk).

The task was structured as in Sebastian *et al*. (2012a, 2012b). In total, 30 cartoons (10 of each condition) were used, presented in sets of six, with a 15-second fixation period between sets. The six cartoons in each set included two cartoons from each condition, which were always yoked together. The order in which the ToM, Affective ToM and PC cartoon pairs in each set were presented was randomized for each participant. Each cartoon exemplar was presented once only.

Each cartoon involved four sequential frames. The first screen (3 seconds) displayed ‘What happens next?’ This was followed by three sequentially presented story frames, each presented for 2 seconds. The final screen, displayed for 5 seconds, showed a choice of two possible endings for the cartoon. During this time participants made their choice using a keypad. The inter-stimulus interval was 1 second, so each trial lasted 15 seconds in total.

For ToM cartoons, selecting the correct ending required understanding behaviour based on intentions (e.g. using an umbrella to help reach a door handle). PC cartoons required an understanding of cause and effect reasoning (e.g. understanding that a hat cannot blow against the wind). Affective ToM cartoons involved understanding behaviour based on emotion.

### fMRI data acquisition

Images were acquired using a Siemens Avanto 1.5-T MRI scanner. These included a 5.5-minute T1 weighted structural scan, and 184 multislice T2-weighted echo planar volumes with blood oxygenation level-dependent contrast, taken during 1 9-minute run of the cartoon task. Acquisition parameters were: 35 2 mm slices with a 1 mm gap; echo-time = 50 milliseconds; repetition time = 2975 milliseconds; slice tilt = −30 (T  >  C): flip angle = 90^o^; field of view = 192 mm; matrix size = 64 × 64. Fieldmaps were also obtained and used to adjust functional scans for deformations due to magnetic field in-homogeneities during pre-processing.

### fMRI data analysis

SPM8 (http://www.fil.ion.ucl.ac.uk/spm) running in MATLAB R2007b was used to analyse imaging data. Five volumes were removed from the beginning of the sequence and two from the end, to allow for T1 equilibration and the final ‘Thank you’ screen. Voxel displacement maps were created from the fieldmaps for each participant, and used during the realign and unwarp stage of pre-processing. Images were normalized using warps created from segmented structural scans which had been co-registered to the functional data, written with a voxel size of 2 × 2 ×  2 mm, and smoothed using an 8-mm Gaussian kernel. Images showing visible motion-related distortions were removed and interpolated using adjacent scans to prevent distortion of the between-subjects mask. Interpolated scans were then regressed out in the first-level design matrix. Movement artefacts were detected in 17 participants (TD: *N *=* *3, ASD: *N *=* *7, CP/HCU: *N *=* *7), and always constituted less than 10% of each subject's data.

The first-level design matrix deconstructed the time series into segments corresponding to each of the three cartoon types (11 seconds each), periods of fixation (15 seconds) or instructions (3 s) and inter-stimulus intervals (1 s). The regressors were modelled as box-car functions and convolved with a canonical hemodynamic response function. Realignment parameters and interpolated scans were also included as regressors. The contrast of interest (ToM > PC) was estimated in each participant. Contrast images were then used in second-level analyses; with group (TD; CP/HCU; ASD) as the between-subjects factor in a one-way ANOVA.

Main effects for ToM > PC were thresholded at *p *<* *.05 family-wise error (FWE) corrected at peak level. For interactions with group, a priori regions of interest (bilateral medial/ventromedial prefrontal cortex, temporal poles and pSTS/TPJ) were defined using the aal atlas in the WFU Pickatlas toolbox for SPM (Maldjian, Laurienti, Kraft & Burdette, 2003; Tzourio-Mazoyer, Landeau, Papathanassiou, Crivello, Etard, Delcroix, Mazoyer & Joliot, 2002). Results within these ROIs were thresholded at *p *<* *.05, small-volume FWE-corrected at the peak level within each ROI. The Marsbar toolbox (Brett, Jean-Luc, Valabregue & Poline, 2002) was used to extract mean responses across significant clusters within ROIs for plotting purposes, and for conducting correlational analyses within the ASD group. An exploratory whole brain analysis was also conducted, with results reported at *p *<* *.001, k > 5, uncorrected (see Table S3).

## Results

### Behavioural data

Mean error rates and reaction times are displayed in Table2. For error rates, a Group (TD, CP/HCU, ASD) × Condition (ToM, PC) mixed model ANOVA revealed no significant main effect of condition, *F*(1, 45) =.78, *p *=* *.381, η^2^_*partial*_ =.017. There was no significant main effect of group, *F*(2, 45) = 1.95, *p *=* *.154, η^2^_*partial*_ =.080, or Group × Condition interaction, *F*(2, 45) =.09, *p *=* *.917, η^2^_*partial*_ =.004, which would have complicated interpretation of fMRI data. For reaction times, there was a significant main effect of condition, *F*(1, 45) = 5.68, *p *=* *.021, η^2^_*partial*_ =.112. Post-hoc analysis revealed significantly faster responses to PC versus ToM across all groups, *t*(47) = 2.40, *p *=* *.02, Cohen's *d *=* *.75. There was no main effect of group, *F*(2, 45) = 2.52, *p *=* *.092, η^2^_*partial*_ =.101, nor Group × Condition interaction, *F*(2, 45) =.64, *p *=* *.532, η^2^_*partial*_ =.028.

**Table 2:** Behavioural data: mean (SD). Abbreviations: RT = reaction time; msec = milliseconds; ns = not significant

Behavioural Data	Typically developing (*N* = 16)	CP/HCU (*N* = 16)	ASD (*N* = 16)	Main effect of group	Group × condition
ToM errors (%)	15.63 (12.09)	10.00 (11.55)	10.00 (10.33)	*ns*	*ns*
Physical Causality (PC) errors (%)	12.50 (12.91)	8.75 (8.06)	8.75 (8.06)		
ToM RT (msec)	2080 (465)	2393 (405)	2313 (499)	*ns*	*ns*
Physical Causality (PC) RT (msec)	1965 (404)	2224 (349)	2270 (465)		

### ToM > PC: main effects

**Figure 1:**
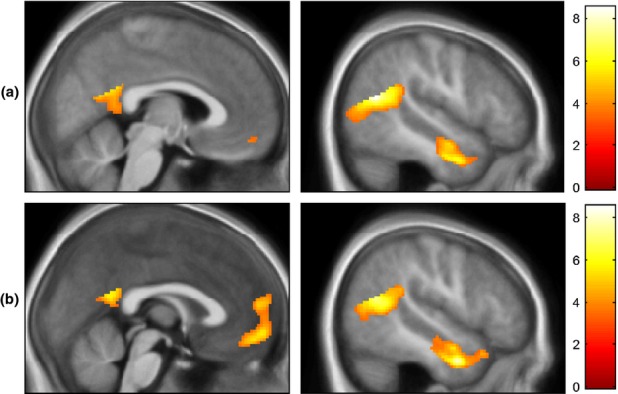
Main effects for ToM > PC for (a) All three groups combined and (b) the TD and CP/HCU groups only. Results are overlaid on an average structural for all participants. Results are shown at a threshold of p < .001, k > 5, uncorrected. Colour-bar represents t-values.

Regions surviving whole brain FWE-correction at *p *<* *.05 for ToM > PC are detailed in Table3. Significant clusters were detected in the bilateral TPJ, extending to occipito-temporal cortex, bilateral temporal poles, left parahippocampal gyrus and posterior cingulate cortex. No responses were observed in the medial prefrontal cortex (mPFC) when data from all three groups were included, although at an uncorrected threshold of *p *<* *.001, k > 5, a cluster was seen in ventromedial PFC (MNI: x = 4, y = 50, z  =  −14, k = 15; Figure1a). When main effects were re-run with only TD and CP/HCU groups, a cluster in the anterior rostral mPFC (MNI: x = 6, y = 54, z = 14; k = 5) survived FWE-correction at *p *<* *.05 (Figure1b). Reverse contrasts are included in Table S2.

**Table 3:** Regions showing a main effect at p < .05 with FWE correction at the peak level for ToM > PC. Abbreviations: TPJ = temporoparietal junction; BA = Brodmann area; k  =  cluster size; ext. = extending to. Where more than one BA is shown, the peak voxel falls in the first BA but the cluster extends to the others listed

			Peak voxel (MNI)				
Brain region ToM > PC	BA	L/R	x	y	z	k	*z*-value
TPJ, ext. to occipitotemporal cortex	39, 22, 19	R	50	−58	18	424	6.48
TPJ, ext. to occipitotemporal cortex	22	L	−36	−54	15	188	6.12
	39, 19	L	−44	−60	18		6.05
Parahippocampal gyrus	37	L	−30	−42	−8	47	5.68
Temporal pole, ext. to fusiform gyrus	21	R	58	4	−20	181	5.64
	21, 20	R	54	0	−26		5.64
	20	R	48	−8	−26		4.92
Temporal pole	21	L	−60	−2	−20	19	5.21
Posterior cingulate cortex/ precuneus	30	L	−14	−58	16	13	5.03
Posterior cingulate cortex	23, 30, 31	Midline	0	−50	22	6	4.79

### ToM > PC: interactions with group

We first examined whether TD and CP/HCU responses differed within ROIs and across the whole brain for ToM > PC. As predicted, no differences between groups survived small-volume FWE-correction within ROIs, and only one small cluster (middle frontal gyrus, k = 6) was significant in the whole brain at a liberal threshold of *p *<* *.001, k > 5 (Table S3). Because no regions, bar this one area, showed group differences at corrected or liberal thresholds for either TD > CP/HCU or CP/HCU > TD, we compared ASD against TD and CP/HCU groups combined (set up in contrasts as 0.5 (TD) 0.5 (CP/HCU) and −1 (ASD)).

Within a priori ROIs, three clusters in medial/ventromedial PFC showed a Group × Condition interaction in the predicted direction (TD = CP/HCU > ASD for ToM > PC) at *p *<* *.05, small-volume FWE-corrected (Table4). No other ROIs yielded significant results.

**Table 4:** Clusters within masked regions showing a Condition × Group interaction at p < .05, SVC-FWE corrected. BA = Brodmann area, k  =  cluster size, SVC-FWE = small volume family-wise error corrected

			Peak voxel (MNI)						
Region included in mask	BA	L/R	x	y	z	k	z-value	SVC-FWE peak p-value	Cohen's d
TD + CP/HCU > ASD									
Medial prefrontal cortex	10	L	−8	60	8	3	3.91	.035	1.47
	10	L	−12	54	16	1	3.81	.048	1.43
Ventromedial prefrontal cortex	10	L	−4	60	−4	2	3.56	.035	1.31

**Figure 2:**
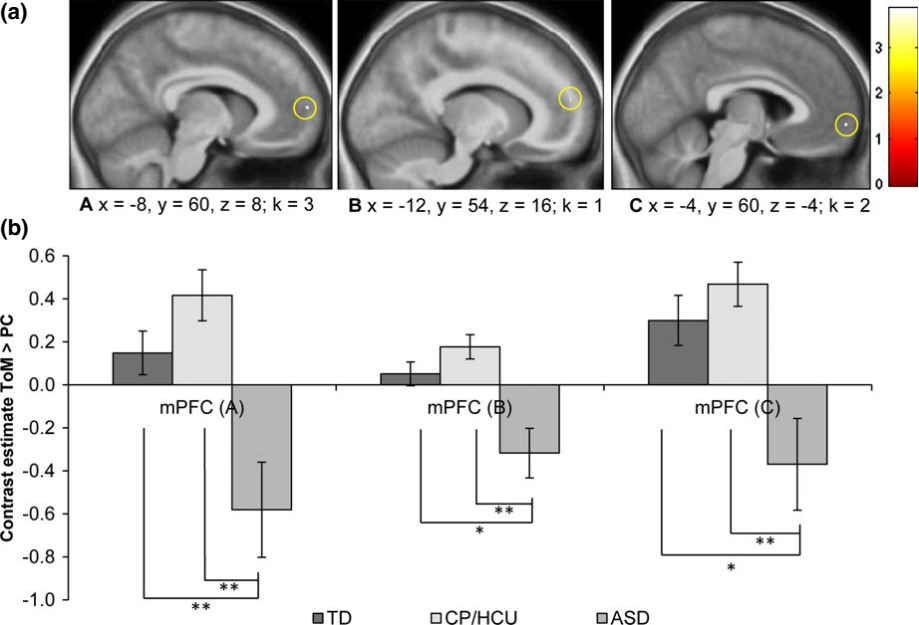
(a) Clusters surviving small-volume corrected FWE thresholds for the condition × group interaction (TD = CP/HCU > ASD). Colour bars represent t-values. (b) Parameter estimates averaged across voxels in the cluster using Marsbar (Maldijan et al., 2003). A: (x  =  −8, y = 60, z = 8), k = 3; B: (x  =  −12, y = 54, z = 15), k = 1, C: (x  =  −4, y = 60, z  =  −4), k = 2. Error bars indicate standard errors. Analyses indicated significant differences between TD vs. ASD and CP/HCU vs. ASD (* = *p* < .05; ** = *p* < .005).

Parameter estimates were extracted by averaging across voxels in the clusters surviving small-volume FWE-correction using Marsbar. Figure2 illustrates that neural responses of children with ASD were significantly reduced compared with TD and CP/HCU children. Results for group comparisons were very similar when symptom scores which significantly differed between groups (ADHD, depression and anxiety) or IQ were included as covariates.

### Correlational analyses in the ASD group

**Figure 3:**
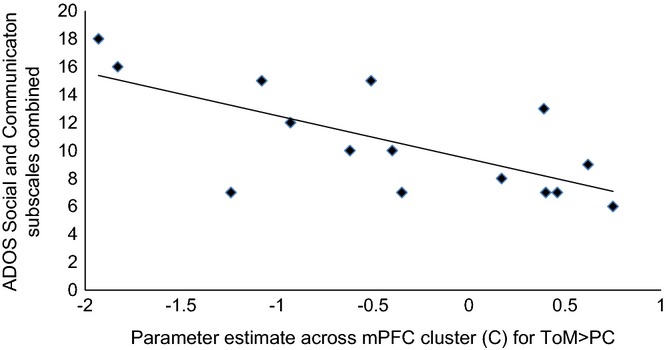
Correlation between functional response in the mPFC (cluster C in Figure2) for ToM > PC and combined ADOS Social and Communication subscales (*r*  =  −.69, *p* = .004).

To explore whether differences in functional response for the clusters exhibiting Group × Condition interactions were also associated with severity of symptoms within the ASD group, we investigated the relationship between parameter estimates across clusters for ToM > PC and combined social and communication subscales of the ADOS. An inverse correlation was observed between mPFC response (cluster C) and social and communication subscales combined (*r * =  −.69, *p *=* *.004; Figure3). This result survives correction for multiple comparisons across the three correlations performed. Results for the other mPFC clusters were in the predicted direction, but not significant.

**Figure 4:**
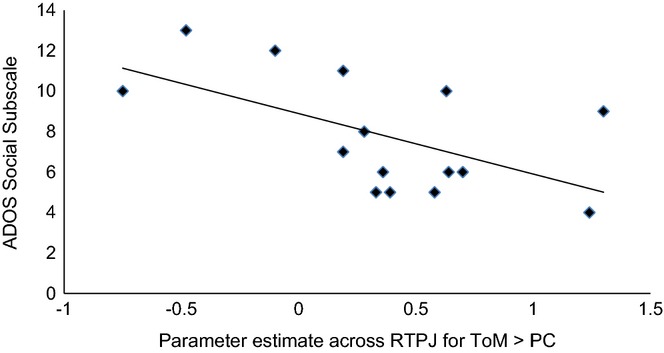
Correlation between functional response in the RTPJ for ToM > PC and ADOS Social subscale (*r*  =  −.57, *p* = .027).

Bilateral TPJ was also of a priori interest because of its relevance to ToM (e.g. Saxe & Kanwisher, 2003), and previous reports of correlations between functional response during ToM and ASD symptoms (Kana *et al*., 2009; Lombardo *et al*., 2011). Marsbar was used to extract mean contrast estimates within the ASD group for right and left TPJ clusters showing a main effect of ToM > PC at whole brain FWE-corrected levels across the whole sample. There was no significant correlation between ADOS social and communication subscales combined; however, the social subscale was inversely correlated with rTPJ functional response (*r * =  −.57, *p *=* *.027; Figure4). Cook's distance and leverage values were within acceptable limits.

## Discussion

This study is the first to compare the neural basis of ToM processing in two groups of children who present with marked social deficits – those with ASD and those with CP/HCU. Compared to CP/HCU children, children with ASD exhibited reduced activation in the medial prefrontal cortex during ToM processing. The same pattern was observed when ASD children were compared to TD controls. There were no differences across ROIs between TD and CP/HCU groups. These findings indicate that whilst individuals with ASD show atypical neural processing, CP/HCU do not have a functional neural impairment during ToM. This is consistent with neurocognitive models that identify a core deficit in ASD as ‘knowing’ about others' mental states (Baron-Cohen *et al*., 1985). By contrast, this process appears spared in CP/HCU children who are instead characterized by not caring about others' feelings (Sebastian *et al*., 2012a; Jones *et al*., 2011; Blair, 2005).

Reduced mPFC responses in ASD compared to TD and CP/HCU children are in line with previous studies in both adult (Castelli *et al*., 2002; Happé *et al*., 1996; Kana *et al*., 2009; Kennedy & Courchesne, 2008; Murdaugh *et al*., 2012; Watanabe *et al*., 2012) and developmental (Carter *et al*., 2012; Wang *et al*., 2007) ASD samples, even when, as in the present study, behavioural responses do not differ. The mPFC is considered of central importance in ToM. Amodio and Frith (2006) propose that this region could be implicated in our ability to reason about other minds in the abstract, and integrate knowledge about their attributes with ongoing processing of intentions. In the ROI analysis, all clusters that survived correction were in the left hemisphere. This was surprising given that neuropsychological studies have implicated the right hemisphere in ToM (e.g. Brownell, Griffin, Winner, Friedman & Happé, 2000). However, lowering the threshold revealed that the mPFC cluster exhibiting reduced responses in ASD vs. TD and CP/HCU children did extend to the right hemisphere.

Extracted parameter estimates from mPFC illustrate that whilst TD and CP/HCU groups exhibited an increase in functional response during ToM relative to PC, the ASD group displayed a relatively reduced response. Though most studies have reported group differences in ASD reflecting a lack of differential response for ToM compared to baseline, one previous study also reported a relative deactivation for this contrast (in the RTPJ when making mentalistic judgements about the Queen's views; Lombardo *et al*., 2011). Other studies have not provided parameter estimates for contrasts, making it difficult to explore relative deactivation in ASD. Differences in the paradigms used (e.g. whether or not the PC condition involved agents) could also contribute to differences in results across studies (Castelli *et al*., 2002). One possible explanation for relative reductions in functional response for ToM may be that the presence of people in the logically engaging PC condition provoked more social processing than the ToM condition in the ASD group. Alternatively, mPFC responses could reflect domain general computation selectively engaged in ASD when processing cause and effect related to physical events.

Within the ASD group, autistic symptoms correlated significantly with functional response for ToM > PC in the most ventral of three clusters exhibiting a Group × Condition interaction in the medial prefrontal cortex. As socio-communicative impairment increased, participants' mPFC response for ToM > PC decreased, in line with similar reports in previous studies (e.g. Wang *et al*., 2007; Watanabe *et al*., 2012). This finding is consistent with group-level differences, and suggests that severity of autistic symptoms is related to degree of atypicality of social processing in mPFC – although causal direction cannot be assumed.

A further a priori region of interest was the TPJ, strongly implicated in ToM (Gweon, Dodell-Feder, Bedny & Saxe, 2012; Saxe & Kanwisher, 2003) and showing reduced responses during ToM in ASD compared with controls in previous studies (Kana *et al*., 2009; Lombardo *et al*., 2011). Consistent with these reports, RTPJ response for ToM > PC showed a negative relationship with ADOS social symptoms. However, no RTPJ clusters exhibited a Group × Condition interaction at small-volume FWE thresholds, or even at a liberal threshold (*p *<* *.001, k > 5; Table S3), in contrast to previous studies in adults (Lombardo *et al*., 2011).

In conclusion, ASD appears characterized by attenuated mPFC activation during ToM processing. By contrast, CP/HCU children show typical patterns of neural response during ToM processing, comparable to that seen in controls. Given the small sample size, these results should be considered preliminary until replicated in a larger population. Future studies could also explore differences in functional or effective connectivity across the social brain network in these groups. Given the need for methods to differentiate ASD from other clinical populations, including CP/HCU, these findings suggest that sensitive indicators of ToM could assist in differentiating these groups in the clinic.

## Supporting Information

**Table S1.**Diagnostic information for the autism spectrum disorder (ASD) group. Abbreviations: ADOS = autism diagnostic observational schedule, ADI-R = Autism Diagnostic Interview Revised; SCQ = Social and Communication Questionnaire, soc = social, comm = communication, RRBI= rigid and repetitive behaviours and interests. N/A = Not applicable; OCD = obsessive compulsive disorder. Best available instrument refers to whether a diagnostic interview was available (ADI or 3Di), as opposed to the SCQ questionnaire. **Table S2**. Whole brain main effects (across group) of the reverse contrast Physical Causality>Theory of Mind (PC>ToM). Results are thresholded at *p* <.05 FWE-corrected at the peak level across the whole brain. Abbreviations: BA = Brodmann area; k = cluster size. **Table S3**. Regions showing a Condition x Group interaction for ToM > PC across the whole brain at *p* <.005 uncorrected, k > 10. BA = Brodmann area; k = cluster size; TD = typically developing controls; CP/HCU = conduct problem with high callous-unemotional traits; ASD = autism spectrum disorder.
