# Supplementary material for: Neural bases of Theory of Mind in children with autism spectrum disorders and children with conduct problems and callous-unemotional traits
Source: Dev Sci. 2014 Mar 17;17(5):786–96. doi: 10.1111/desc.12167 (PMC4316185; doi:10.1111/desc.12167)
Supplement: Supplementary file 1 — Table S1.Diagnostic information for the autism spectrum disorder (ASD) group. Abbreviations: ADOS = autism diagnostic observational schedule, ADI-R = Autism Diagnostic Interview Revised; SCQ = Social and Communication Questionnaire, soc = social, comm = communication, RRBI= rigid and repetitive behaviours and interests. N/A = Not applicable; OCD = obsessive compulsive disorder. Best available instrument refers to whether a diagnostic interview was available (ADI or 3Di), as opposed to the SCQ questionnaire. Table S2. Whole brain main effects (across group) of the reverse contrast Physical Causality>Theory of Mind (PC>ToM). Results are thresholded at p <.05 FWE-corrected at the peak level across the whole brain. Abbreviations: BA = Brodmann area; k = cluster size. Table S3. Regions showing a Condition x Group interaction for ToM > PC across the whole brain at p <.005 uncorrected, k > 10. BA = Brodmann area; k = cluster size; TD = typically developing controls; CP/HCU = conduct problem with high callous-unemotional traits; ASD = autism spectrum disorder. [file desc0017-0786-sd1.docx]

|  |  | Observed Characteristics | | | | | | Parent-Reported Characteristics | | | |
| --- | --- | --- | --- | --- | --- | --- | --- | --- | --- | --- | --- |
| **ID** | **Diagnosis** | **ADOS Soc**  ***Cut-off = 4*** | **ADOS Comm *Cut-off = 2*** | **ADOS RRBI** | **ADOS Total *Cut-off = 7*** | **ADOS Classi-fication** |  | | **Best Instru-ment** | **Designation** |  |
| 1 | Asperger’s syndrome | 5 | 3 | 2 | 8 | ASD |  | | 3Di | ASD (comm only) |  |
| 2 | Autism/Asperger's syndrome | 9 | 6 | 2 | 15 | Autism |  | | ADI | ASD (soc & comm) |  |
| 3 | Autism/Asperger's syndrome; OCD | 13 | 5 | 0 | 18 | Autism |  | | SCQ | Above risk cut-off |  |
| 4 | Autism/Asperger's syndrome; dyslexia | 12 | 4 | 2 | 16 | Autism |  | | 3Di | Asperger |  |
| 5 | Asperger's syndrome | 6 | 1 | 1 | 7 | ASD |  | | ADI | ASD (soc & comm) |  |
| 6 | Autism/Asperger's syndrome | 5 | 2 | 3 | 7 | ASD |  | | 3Di | Asperger |  |
| 7 | Autism | 7 | 2 | 0 | 9 | ASD |  | | SCQ | Above risk cut-off |  |
| 8 | Autism; dyspraxia | 11 | 4 | 6 | 15 | Autism |  | | SCQ | Above risk cut-off |  |
| 9 | Autism/Asperger's syndrome | N/A | N/A | N/A | N/A | N/A |  | | 3Di | ASD (comm & RRBI) |  |
| 10 | Autism; dyspraxia | 8 | 2 | 0 | 10 | Autism |  | | 3Di | Asperger |  |
| 11 | Autism/Asperger's syndrome | 6 | 1 | 0 | 7 | ASD |  | | ADI | ASD (soc & comm) |  |
| 12 | Autism/Asperger's syndrome | 4 | 2 | 4 | 6 | None |  | | 3Di | Asperger |  |
| 13 | Asperger's syndrome | 6 | 4 | 1 | 10 | Autism |  | | SCQ | Above risk cut-off |  |
| 14 | Asperger's syndrome | 10 | 3 | 1 | 13 | Autism |  | | SCQ | Above risk cut-off |  |
| 15 | Autism; dyslexia | 5 | 2 | 0 | 7 | ASD |  | | SCQ | Above risk cut-off |  |
| 16 | Autism | 10 | 2 | 0 | 12 | Autism |  | | SCQ | Above risk cut-off |  |

**Supplementary Materials for ‘Neural bases of Theory of Mind in children with autism spectrum disorders and children with conduct problems and callous-unemotional traits’.**

**Supplementary Tables**

*Supplementary Table 1*

*Supplementary Table 1:* Diagnostic information for the autism spectrum disorder (ASD) group. Abbreviations: ADOS = autism diagnostic observational schedule, ADI-R = Autism Diagnostic Interview Revised; SCQ = Social and Communication Questionnaire, soc = social, comm = communication, RRBI= rigid and repetitive behaviours and interests. N/A = Not applicable; OCD = obsessive compulsive disorder. Best available instrument refers to whether a diagnostic interview was available (ADI or 3Di), as opposed to the SCQ questionnaire.

*Supplementary Table 2*

| **Brain region**  **PC> ToM** | **BA** | **L/R** | **Peak voxel (MNI)**  **x y z** | | | **k** | **z** |
| --- | --- | --- | --- | --- | --- | --- | --- |
| Postcentral Gyrus | 3 | R | 62 | -16 | 28 | 276 | 6.97 |
| Precentral Gyrus | 44 | R | 52 | 10 | 16 | 399 | 6.48 |
| Inferior Frontal Gyrus | 9 | R | 50 | 6 | 26 |  | 5.54 |
| Inferior Frontal Gyrus | 46 | R | 42 | 38 | 12 | 377 | 6.40 |
| Inferior Frontal Gyrus | 10 | R | 46 | 46 | 0 |  | 5.37 |
| Inferior Frontal Gyrus | 46 | L | -40 | 38 | 12 | 120 | 5.78 |
| Middle Temporal Gyrus | 21 | R | 64 | -44 | -8 | 127 | 5.50 |
| Lingual Gyrus | 18 | R | 18 | -74 | -12 | 114 | 5.44 |
| Claustrum |  | L | -38 | -2 | 2 | 12 | 5.00 |
| Middle Occipital Gyrus | 19 | R | 50 | -56 | -10 | 13 | 4.76 |
| Middle Frontal Gyrus | 11 | R | 20 | 32 | -14 | 1 | 4.75 |
| Postcentral Gyrus | 1 | L | -62 | -18 | 30 | 5 | 4.74 |
| Inferior Frontal Gyrus | 46 | L | -46 | 42 | 4 | 8 | 4.68 |
| Precentral Gyrus | 44 | L | -54 | 12 | 10 | 1 | 4.64 |
| Lingual Gyrus | 18 | L | -14 | -82 | -14 | 1 | 4.62 |
| Precentral Gyrus | 44 | L | -52 | 10 | 12 | 1 | 4.62 |

*Supplementary Table 2:* Whole brain main effects (across group) of the reverse contrast Physical Causality>Theory of Mind (PC>ToM). Results are thresholded at *p* < .05 FWE-corrected at the peak level across the whole brain. Abbreviations: BA = Brodmann area; k = cluster size.

*Supplementary Table 3*

| **Brain Region**  **ToM>PC** | **BA** | **L/R** | **Peak voxel (MNI)**  **x y z** | | | **k** | **z** |
| --- | --- | --- | --- | --- | --- | --- | --- |
| **TD vs. CP/HCU** |  |  |  |  |  |  |  |
| Middle frontal gyrus | 9 | R | 34 | 18 | 34 | 6 | 3.33 |
| **CP/HCU vs. TD** |  |  |  |  |  |  |  |
| No suprathreshold voxels |  |  |  |  |  |  |  |
| **TD+CP/HCU vs. ASD** |  |  |  |  |  |  |  |
| Anterior-rostral medial PFC ext. to anterior cingulate | 10 | L | -8 | 60 | 8 | 484 | 3.91 |
|  |  | R | 2 | 54 | 12 |  | 3.83 |
|  |  | L | -12 | 54 | 16 |  | 3.81 |
| Anterior cingulate | 32 | R | 20 | 36 | 14 | 12 | 3.69 |
| Brainstem (Midbrain)/ Thalamus |  | R | 4 | -26 | 0 | 27 | 3.63 |
| Brainstem (Midbrain)/ Thalamus |  | L | -4 | -32 | -4 |  | 3.16 |
| Temporal pole | 38 | R | 48 | 20 | -20 | 16 | 3.43 |
| Lentiform nucleus/ Putamen |  | L | -18 | 10 | -14 | 5 | 3.35 |
| Thalamus (pulvinar)/ Caudate tail |  | L | -16 | -28 | 18 | 7 | 3.34 |
| Superior frontal gyrus | 9 | R | 4 | 58 | 28 | 19 | 3.23 |
| Middle frontal gyrus | 10 | L | -44 | 50 | -2 | 19 | 3.21 |
| **ASD vs. TD+CP/HCU** |  |  |  |  |  |  |  |
| Cingulate gyrus | 24 | L | -8 | -4 | 28 | 12 | 3.44 |

*Supplementary Table 3:* Regions showing a Condition x Group interaction for ToM > PC across the whole brain at *p* < .005 uncorrected, k > 10. BA = Brodmann area; k = cluster size; TD = typically developing controls; CP/HCU = conduct problem with high callous-unemotional traits; ASD = autism spectrum disorder.
